# Adverse Cardiovascular Effects of Phenylephrine Eye Drops Combined With Intravenous Atropine

**DOI:** 10.3389/fphar.2020.596539

**Published:** 2021-01-27

**Authors:** Qingyu Li, Jianxin Pang, Yang Deng, Shaochong Zhang, Yong Wang, Yang Gao, Xiaoyong Yuan, Yantao Wei, Hongbin Zhang, Junlian Tan, Wei Chi, Wenjun Guo

**Affiliations:** ^1^ State Key Laboratory of Ophthalmology, Zhongshan Ophthalmic Center, Sun Yat-sen University, Guangzhou, China; ^2^ Clinical College of Ophthalmology, Tianjin Medical University, Tianjin, China; ^3^ Guangdong Provincial Key Laboratory of Drug Screening, School of Pharmaceutical Sciences, Southern Medical University, Guangzhou, China; ^4^ Shenzhen Eye Hospital, Shenzhen Eye Institute, Shenzhen Eye Hospital affiliated to Jinan University, School of Optometry, Shenzhen University, Shenzhen, China; ^5^ Foresea Life Insurance Guangzhou General Hospital, Guangzhou, China; ^6^ Department of Medical Research, Liuhuaqiao Hospital, Guangzhou, China

**Keywords:** phenylephrine, atropine, Combination, neostigmine, adverse cardiovascular effects, hypertension emergency

## Abstract

**Background:** Phenylephrine and atropine can cause serious adverse effects when applied in combination. We investigated the effect of phenylephrine eye drops combined with intravenous atropine on the cardiovascular system in patients under general anesthesia undergoing intraocular surgery.

**Methods:** The effects of the drugs were observed through clinical study. Thirteen patients undergoing intraocular surgery under general anesthesia were observed in this study; all were injected intravenously with atropine due to the oculocardiac reflex during surgery. To study the combination of drugs, an *in vivo* study was performed on rats. Seventy-two standard deviation rats that received phenylephrine eye drops and intravenous atropine treatment under general anesthesia were assessed, of which 18 treated with these drugs simultaneously were administered normal saline, neostigmine or esmolol. Blood pressure and heart rate were recorded and analyzed.

**Findings:** The age of the patients ranged from seven to 14 years old with an average age of 10.7 years old, and 11 patients were male. In patients, 5% phenylephrine eye drops combined with intravenous atropine led to a significant heart rate increase and the increase lasted 20 min. The significant increase in diastolic blood pressure and systolic blood pressure lasted for 15 and 25 min, respectively. From five to 25 min after intravenous atropine treatment, the systolic blood pressure and diastolic blood pressure were both more than 20% higher than that at baseline. In rats, the changes in blood pressure and heart rate were independent of the phenylephrine and atropine administration sequence but were related to the administration time interval. The neostigmine group showed a significant decrease in blood pressure after the increase from the administration of phenylephrine and atropine.

**Interpretation:** Phenylephrine eye drops combined with intravenous atropine have obvious cardiovascular effects that can be reversed by neostigmine. This drug combination should be used carefully for ophthalmic surgery, especially in patients with cardio-cerebrovascular diseases.

## Introduction

Agonism of alpha 1-adrenoceptors in the iris dilator leads to mydriasis. Phenylephrine is a selective alpha 1-adrenergic agonist. It can produce prompt and transient mydriasis without increasing the intraocular pressure or affecting the accommodative ability of the eye (cycloplegia) (Esteve-Taboada et al., 2016). Phenylephrine (1%–10%) is often used as a mydriatic agent to facilitate ophthalmic examination and surgery in clinical practice (Stavert et al., 2015). Phenylephrine eye drops can cause systemic side effects, including blood pressure elevation and heart rate alterations, via absorption through the conjunctiva and nasal mucosa (Fraunfelder et al., 2002; Alpay et al., 2010). However, a systematic review and meta-analysis revealed that the systemic side effects of phenylephrine may have been overstated over the years (Stavert et al., 2015). The review showed that 2.5% phenylephrine does not cause clinically significant changes in blood pressure or heart rate in either neonates or adults, and 10% phenylephrine may lead to hemodynamic instability in infants but has no adverse effects in adults due to transient and recoverable changes in blood pressure and heart rate (Stavert et al., 2015). Moreover, these changes in adults are innocuous, similar to circadian or postural blood pressure changes (Stavert et al., 2015).

Ophthalmic surgery operations that stimulate eyeball or ocular tissue can induce the oculocardiac reflex (OCR) directly, and OCR incidence is high during surgery (Aletaha et al., 2016). Serious side effects due to OCR, such as bradycardia, arrhythmia and even cardiac arrest, can occur during eye surgery, especially during corrective strabismus surgery (Deb et al., 2001). Opioids are often used to provide analgesia during the induction and maintenance of general anesthesia (Cravero et al., 2019). The heart rate can be slowed due to the activation of opioid-specific receptors (Chen and Ashburn, 2015). Atropine is an antimuscarinic agent that can compete with acetylcholine for the acetylcholine receptor, thus antagonizing the muscarine-like actions of acetylcholine (Blumenberg, 1987). Atropine is administered as premedication during general anesthesia. Intravenous atropine treatment is often used clinically to inhibit glandular secretion and relieve severe bronchospasm under general anesthesia. More notably, atropine is a very useful drug for preventing or reducing the incidence of sinus bradycardia or bradycardia caused by OCR or opioid applications in eye surgery (Klockgether-Radke et al., 1993; Gilani et al., 2005; Sajedi et al., 2013; Ho et al., 2018). However, atropine cannot totally prevent bradycardia but rather may cause bigeminy and increase ectopic beats, which are more persistent than the OCR (Espahbodi et al., 2015). Generally, atropine is also used as a cycloplegic to temporarily paralyze the accommodation reflex and as a mydriatic to dilate the pupils.

Phenylephrine eye drops and intravenous atropine treatment are often used together in ophthalmic surgeries while patients are under general anesthesia in clinical practice. Although adverse cardiovascular reactions following phenylephrine instillation or intravenous atropine treatment have been reported, the adverse effects and interaction mechanisms of the combination of the two drugs are unclear. Notably, we first noticed a phenomenon in clinical practice where concurrent application of phenylephrine eye drops and intravenous atropine during eye surgery can cause clinically significant increases in heart rate and blood pressure, which could even be life-threatening without intervention. This phenomenon prompted us to think and consult the relevant literature. Keys and Violante reported that subcutaneously injected atropine followed by subcutaneously injected phenylephrine caused a higher blood pressure and heart rate than phenylephrine alone due to atropine enhancing the pressor effect of phenylephrine (Keys and Violante, 1942). Levine and Leenen found that after intravenous atropine treatment the blood pressure responses to intravenous phenylephrine treatment were markedly potentiated in normotensive volunteers (Levine and Leenen, 1992). Fraunfelder and Scafidi found that concomitant use of phenylephrine eye drops and atropine eye drops can cause blood pressure elevation and induce tachycardia in some patients (Fraunfelder and Scafidi, 1978). Tropicamide is a synthetic muscarinic antagonist with actions similar to those of atropine. Co-administration of phenylephrine and tropicamide eye drops may lead to severe adverse effects (Pescina et al., 2017). The compound tropicamide eye drop, a compound preparation containing tropicamide and deoxyepinephrine hydrochloride, is commonly used in China as a routine mydriatic agent for fundus examination and refractive examination (Li et al., 2016). Through a literature review, we also found that some cases of systemic adverse reactions associated with the use of compound tropicamide eye drops have been reported. Sbaraglia et al. reported the occurrence of complications in pediatric patients undergoing ophthalmic surgery after the application of a mydriatic agent of the compound tropicamide and attributed the causes to the alpha 1-adrenergic action of phenylephrine, which we think may dismiss the adverse effects of the combination of the two drugs (Sbaraglia et al., 2014). The mechanism and the solutions to the effects of concurrent use of phenylephrine eye drops and intravenous atropine treatment have not been fully discussed; therefore, ophthalmologists do not pay enough attention to these effects, and similar problems have been occurring in clinical work for a long time. Therefore, through our observation of 13 patients and corresponding animal experiments, we hope to attract more attention from ophthalmologists and anaesthetists to prevent similar problems from occurring in the future. The aim of the study was to assess the adverse cardiovascular effects of the concurrent application of phenylephrine eye drops and intravenous atropine during ophthalmic surgery. Another purpose of this paper was to study the mechanism of adverse cardiovascular effects and the corresponding prevention and treatment interventions.

## Materials and Methods

### Patients and Clinical Observation

This retrospective analysis included 13 patients who received 5% phenylephrine eye drops combined with intravenous atropine administration during intraocular surgery while under general anesthesia at the Zhongshan Ophthalmic Center from 2016 to 2019. The studies were reviewed and approved by Medical Ethics Committee of Zhongshan Ophthalmic Center, Sun Yat-sen University. The age of the patients ranged from seven to 14 years old with an average age of 10.7 years old, and 11 patients were male. The induction of general anesthesia was performed using midazolam (0.03 mg/kg), fentanyl (1 μg/kg), atropine (5 μg/kg), propofol (2–3 mg/kg), and vecuronium (0.6 mg/kg). A tracheal tube was inserted approximately 5 min after induction, and propofol (150–200 μg/kg/minute) and remifentanil (0.2 μg/kg/minute) were used to maintain anesthesia. Eye surgery began approximately 10 min after the instillation of 10–20 mg phenylephrine eye drops. The traction of extraocular muscles or compression to the eyeball can cause a temporary decrease in heart rate, known as OCR. If the heart rate (beats/minute, bpm) decreases by more than 10% from the base heart rate, surgical procedures should be stopped, and the traction of the extraocular muscles or compression of the eyeball should be relaxed (Espahbodi et al., 2015). If the OCR is still not recovered within 20 s, 5 μg/kg atropine should be injected intravenously in routine clinical practice (Espahbodi et al., 2015).

In our study, the heart rate, systolic blood pressure (SBP) and diastolic blood pressure (DBP) of all patients were recorded at different points, namely, before anesthesia, immediately after anesthesia induction, and 5, 10, 15, 20, 25, and 30 min after intravenous atropine treatment.

### Reagents

A 0.9% sodium chloride injection (lot number: C180705D) was purchased from Hubei Kelun Pharmaceutical Co., Ltd (Hubei, China). Pentobarbital sodium (lot number: 4,579/50) was purchased from Tocris Cookson Ltd (Bristol, United Kingdom). The 5% phenylephrine eye drops (lot number: 180605) were purchased from Zhongshan Ophthalmic Center, Sun Yat-sen University (Guangdong, China). Atropine sulfate injection (lot number: 1802024) was purchased from Henan Runhong Pharmaceutical Co., Ltd. (Henan, China). Tetracaine hydrochloride eye drops (lot number: 180524) were purchased from Zhongshan Ophthalmic Center, Sun Yat-sen University (Guangdong, China). Esselor hydrochloride injection (lot number: 7B0062005) was purchased from Qilu Pharmaceutical Co. Ltd (Shandong, China). Neostigmine methylsulfate injection (lot number: 1710605) was purchased from Shanghai Xinyi Jinzhu Pharmaceutical Co., Ltd (Shanghai, China).

### Animals and Study Design

Seventy-two wild-type Sprague-Dawley (SD) rats with body weights ranging from 180 to 250 g were purchased from Hunan SJA Laboratory Animal Co., Ltd (Hunan, China). The use of experimental animals in this study complied with the animal ethics principles, and the protocol was approved by the laboratory animal ethics committee (No. 00201878). Based on previous research and animal welfare, we tried to reduce the number of animals we used as long as we could achieve our experimental objectives. To obtain accurate analysis and minimize the number of experimental animals, a proper number of experimental animals was used. The experimental rats were randomly divided into 12 groups using a random number table, with six rats in each group. Specifically, group A received 5% phenylephrine eye drops only; group B received intravenous atropine administration only; group C received phenylephrine and atropine simultaneously; groups D, E, and F received 5% phenylephrine eye drops first and then received intravenous atropine treatment five, ten, and 15 min later, respectively,; groups G, H, and I received intravenous atropine administration first followed by 5% phenylephrine eye drops 5, 10, and 15 min later, respectively,; group J received phenylephrine and atropine simultaneously followed by intravenous saline treatment 1 min later; and groups K and L received phenylephrine and atropine simultaneously followed by 0.15 mg/kg neostigmine and 1.0 mg/kg esmolol treatment 1 min later, respectively.

The rats were anesthetized with an injection of 1% pentobarbital sodium (50 mg/kg). Before the procedure, all catheters were heparinized with 1000 IU/ml heparin to empty the air in the device. The left common carotid artery was isolated while the rats were in the supine position. Arterial cannulas were placed in the common carotid artery of rats and connected to a physiological monitor. Parameters were measured in this study using the BL-420 F biological signal acquisition and analysis system (Chengdu Taimeng Science and Technology Co. Ltd.). After surface anesthesia of the right eye with tetracaine hydrochloride eye drops, a T-shaped incision of conjunctiva approximately 2 mm away from the corneal rim was created by creating an incision approximately 0.3 cm along the corneal rim and then cutting approximately 0.3 cm away from the cornea. Finally, one 5% phenylephrine eye drop was added to the incision. In addition, 0.06 mg/kg atropine, 0.15 mg/kg neostigmine and 1.0 mg/kg esmolol were administered via tail vein injection. For simultaneous administration of phenylephrine and atropine, the right eye was anesthetized and operated first, followed by 0.06 mg/kg intravenous atropine treatment and the administration of one 5% phenylephrine eye drop.

The operations were performed according to the corresponding time intervals based on different groups. The blood pressure and heart rate were recorded 5 min before any treatment until 40 min after treatment.

### Statistical Analysis

Data are all shown as the mean ± standard deviation (SD). The statistical methods used in this study were an analysis of variance (ANOVA) for the comparison of means among groups and Student's t test for the comparison of means between two groups. SPSS 26.0 (SPSS, lnc., Chicago, IL, United States) was used to analyze the data, and all experiments were blinded. A *p* value was considered statistically significant when it was less than 0.05.

## Results

### Changes in the Heart Rate and Blood Pressure of Patients

**FIGURE 1:**
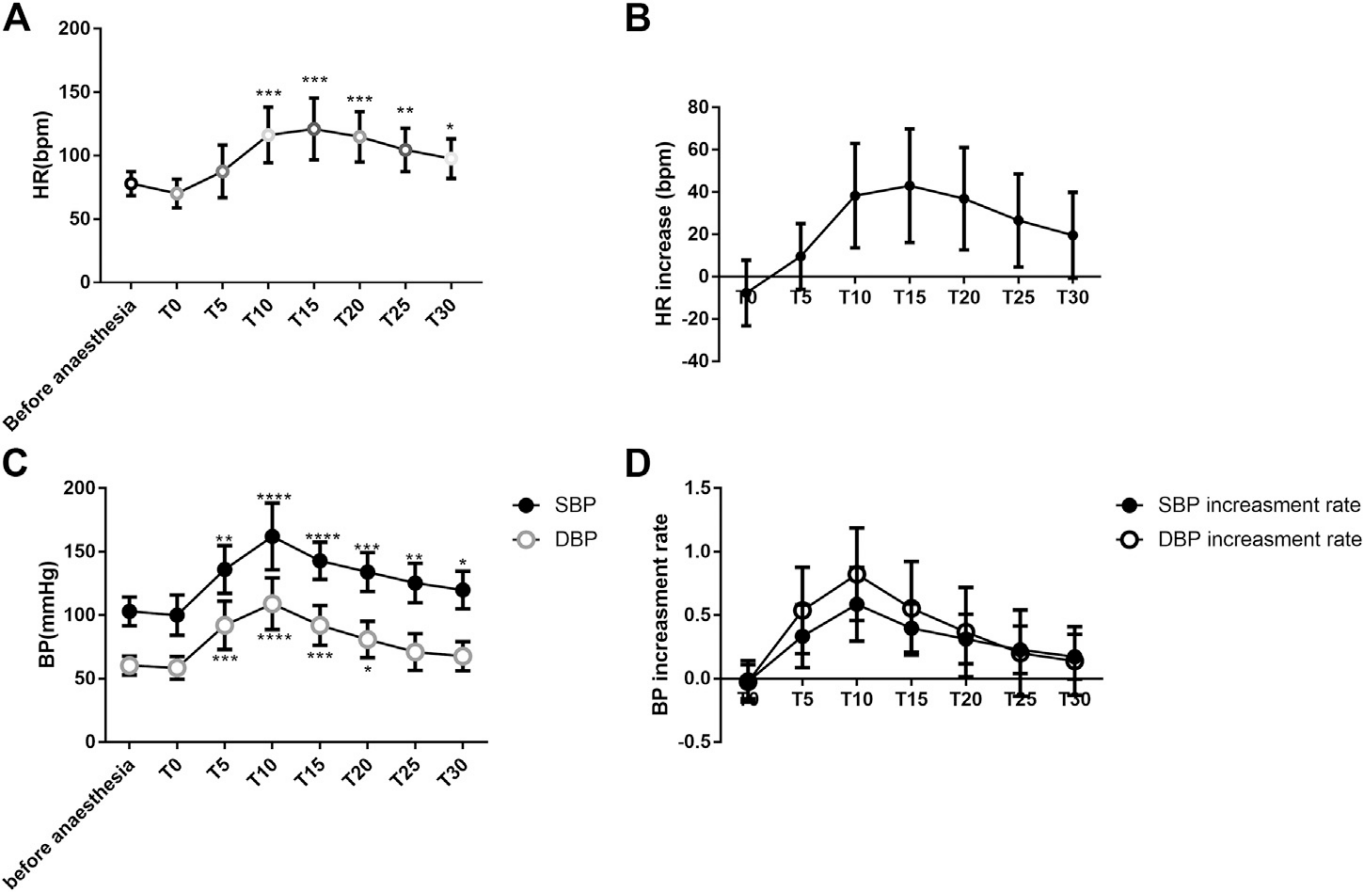
Changes in the heart rate and blood pressure of patients **(A)**. Changes in HR associated with anesthesia and intravenous atropine treatment; **(B)**. Increase in HR associated with anesthesia and intravenous atropine treatment; **(C)**. Changes in blood pressure associated with anesthesia and intravenous atropine treatment; **(D)**. Increase in blood pressure associated with anesthesia and intravenous atropine treatment. Error bars represent the mean ± SD; the comparison was performed using one-way ANOVA. SBP, systolic blood pressure; DBP, diastolic blood pressure; MAP, mean arterial pressure; HR, heart rate. T0, immediately after anesthesia induction; T5, T10, T15, T20, T25, and T30, representing five, ten, 15, 20, 25, and 30 min after intravenous atropine treatment, respectively. N = 13. *p < 0.05, **p < 0.01, and ****p < 0.0001 compared with the before anesthesia group. N = 13.

The heart rate was significantly higher 10 min after intravenous atropine treatment than that at baseline (ANOVA, 95% confidence interval (CI) = −60.39 to −16.22 bpm, *p* = 0.0008). The peak heart rate occurred 15 min after intravenous atropine treatment (ANOVA, 95% CI = −67.11 to −19.04 bpm, *p* = 0.0006) and slowly decreased until 30 min after treatment, but the heart rate was still significantly higher than that at baseline (ANOVA, 95% CI = −37.91 to −1.475 bpm, *p* = 0.0311) (Figure 1A). Topical ocular application of 5% phenylephrine and intravenous treatment with atropine led to a 43.08 ± 26.88 beat/minute increase in heart rate 15 min after intravenous atropine treatment compared with the heart rate before anesthesia (Figure 1B).

At 5 min after intravenous atropine treatment, SBP began to increase and was significantly different from the level before anesthesia (ANOVA, 95% CI = −53.28 to −12.72 mmHg, *p* = 0.0014). The highest SBP occurred 10 min after intravenous atropine treatment (ANOVA, 95% CI = −84.64 to −33.36 mmHg, *p* < 0.0001) and decreased slowly through 30 min after the treatment. SBP was still significantly higher than that before anesthesia (ANOVA, 95% CI = −32.56 to −0.8293 mmHg, *p* = 0.00368). SBP increased from 103.15 ± 11.38 mmHg before anesthesia to 162.15 ± 26.42 mmHg 10 min after intravenous atropine treatment. (Figure 1C). At 5 min after intravenous atropine treatment, DBP was significantly different than the preoperative level (ANOVA, 95% CI = −48.64 to −14.75 mmHg, *p* = 0.0004). The highest DBP appeared 10 min after administration of the medication (ANOVA, 95% CI = −66.8 to −30.43 mmHg, *p* < 0.0001), decreased gradually to the baseline level at 25 min with no difference (ANOVA, 95% CI = −27.22 to 6.144 mmHg, *p* > 0.3724) and remained stable at 30 min after administration of medication. Intravenous atropine treatment caused DBP to increase to the highest level, reaching 109.08 ± 20.41 mmHg from 60.46 ± 7.61 mmHg before anesthesia. (Figure 1C). The significant increase in blood pressure occurred 5 min after intravenous atropine treatment and was sustained for 15 or 25 min (Figure 1C). From five to 30 min after intravenous atropine treatment, the changes in SBP and DBP were all greater than 16.69 ± 17.74 and 7.31 ± 14.60 mmHg, respectively, (Figure 1C). From five to 25 min after intravenous atropine treatment, SBP and DBP were both more than 20% higher than the baseline blood pressure levels (Figure 1D).

### Changes in the Blood Pressure and Heart Rate of Rats

**FIGURE 2:**
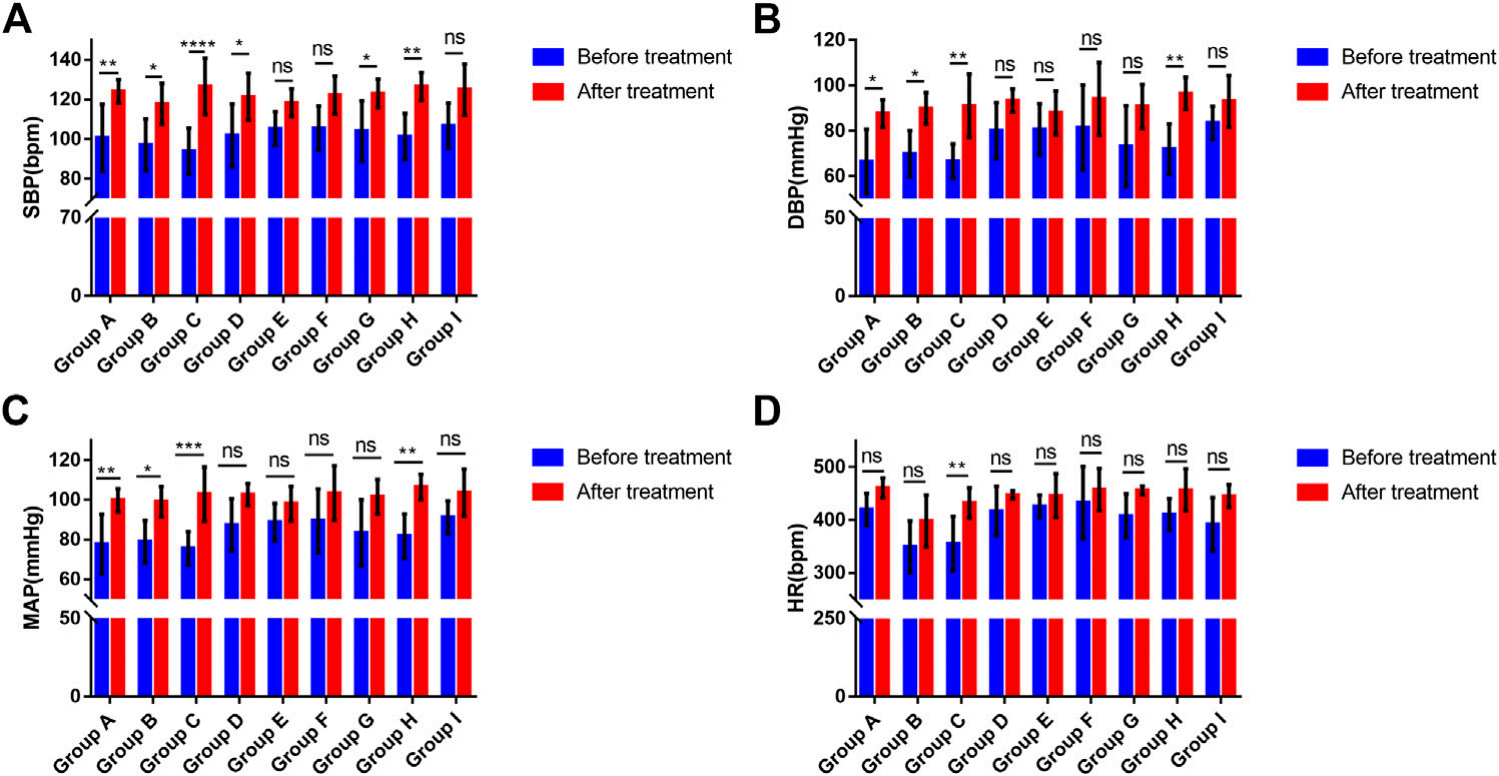
Changes in the blood pressure and heart rate of rats **(A)**. Changes in SBP associated with phenylephrine eye drops and (or) intravenous atropine treatment; **(B)**. Changes in DBP associated with phenylephrine eye drops and (or) intravenous atropine treatment; **(C)**. Changes in MAP associated with phenylephrine eye drops and (or) intravenous atropine treatment; **(D)**. Changes in HR associated with phenylephrine eye drops and (or) intravenous atropine treatment. Error bars represent the mean ± SD. SBP, systolic blood pressure; DBP, diastolic blood pressure; MAP, mean arterial pressure; HR, heart rate. Before treatment, blood pressure and heart rate before the administration of phenylephrine and (or) atropine. After treatment, the highest blood pressure and heart rate during the observation period. Group A received phenylephrine eye drops. Group B received intravenous atropine treatment. Group C received phenylephrine and atropine simultaneously. Groups D, E, and F received phenylephrine eye drops first and then intravenous atropine treatment five, ten, and 15 min later, respectively. Groups G, H, and I received intravenous atropine treatment first followed by phenylephrine eye drops five, ten, and 15 min later, respectively. N = six.

Under anesthesia, no significant differences were found in SBP, DBP, mean arterial pressure (MAP), or heart rate among the groups (ANOVA, F = 0.6178, *p* = 0.7582; ANOVA, F = 1.6, *p* = 0.1519; ANOVA, F = 1.241, *p* = 0.2984; ANOVA, Bartlett's statistic (corrected) = 8.33, *p* = 0.4019) (Figure 2). The blood pressure and heart rate of the rats increased to a certain extent after the administration of phenylephrine or atropine alone (Figure 2). After applying phenylephrine 0, five, ten, and 15 min later, the combined use of intravenous atropine resulted in elevated blood pressure and heart rate compared with the values prior to the administration of medication (Figure 2). After intravenous atropine treatment five, ten, and 15 min later, the phenylephrine eye drops also caused elevated blood pressure and heart rate compared with the values prior to the administration of medication (Figure 2). However, the changes in blood pressure and heart rate reached their peak when phenylephrine and atropine were administered simultaneously (Table 1). The increases in SBP, DBP, and MAP were 32.62 ± 13.58, 24.44 ± 18.18, and 27.28 ± 16.01 mmHg, respectively, and the increase in heart rate was 77.17 ± 57.72 bpm following the administration of both drugs simultaneously (Table 1). With the increase in time interval, the changes in blood pressure and heart rate started to decrease gradually (Table 1, Figure2). The changes in blood pressure and heart rate were independent of the sequence of administration of phenylephrine and atropine but related to the time interval between them. When the time interval between the administration of these two drugs was ten or 15 min, the increases in blood pressure and heart rate were the smallest (Table 1, Figure 2). The results of this experiment suggest that the combined application of phenylephrine and atropine has a synergistic effect of increasing blood pressure and heart rate.

**TABLE 1:** Variations in BP and HP after phenylephrine eye drop and intravenous atropine administration in rats.

	SBP (mmHg)	DBP (mmHg)	MAP (mmHg)	HR (bpm)
Group A	23.51 ± 16.20	21.17 ± 14.25	21.96 ± 14.73	40.33 ± 44.58
Group B	20.60 ± 10.66	19.98 ± 6.81	20.19 ± 7.43	48.67 ± 59.76
Group C	32.62 ± 13.58	24.44 ± 18.18	27.28 ± 16.01	77.17 ± 57.72
Group D	19.58 ± 11.22	13.21 ± 10.53	15.22 ± 10.67	30.33 ± 45.17
Group E	13.14 ± 8.73	7.34 ± 3.38	9.27 ± 4.63	20.50 ± 40.32
Group F	16.68 ± 8.45	12.61 ± 9.14	13.97 ± 8.45	24.83 ± 33.98
Group G	19.01 ± 10.29	17.67 ± 12.68	18.11 ± 11.19	48.00 ± 40.35
Group H	25.20 ± 15.32	24.59 ± 16.84	24.79 ± 16.25	46.00 ± 30.38
Group I	18.25 ± 7.61	9.52 ± 7.34	12.43 ± 7.30	53.33 ± 59.60

Error bars represent the mean ± SD. SBP, systolic blood pressure; DBP, diastolic blood pressure; MAP, mean arterial pressure; HP, heart rate. Group A received phenylephrine eye drops. Group B received intravenous atropine treatment. Group C received phenylephrine and atropine simultaneously. Groups D, E, and F received phenylephrine eye drops first and then intravenous atropine treatment five, ten, and 15 min later, respectively. Groups G, H, and I received intravenous atropine treatment first followed by phenylephrine eye drops five, ten, and 15 min later, respectively. N = six.

### Effects on Blood Pressure and Heart Rate in Rats After Neostigmine Treatment

**FIGURE 3:**
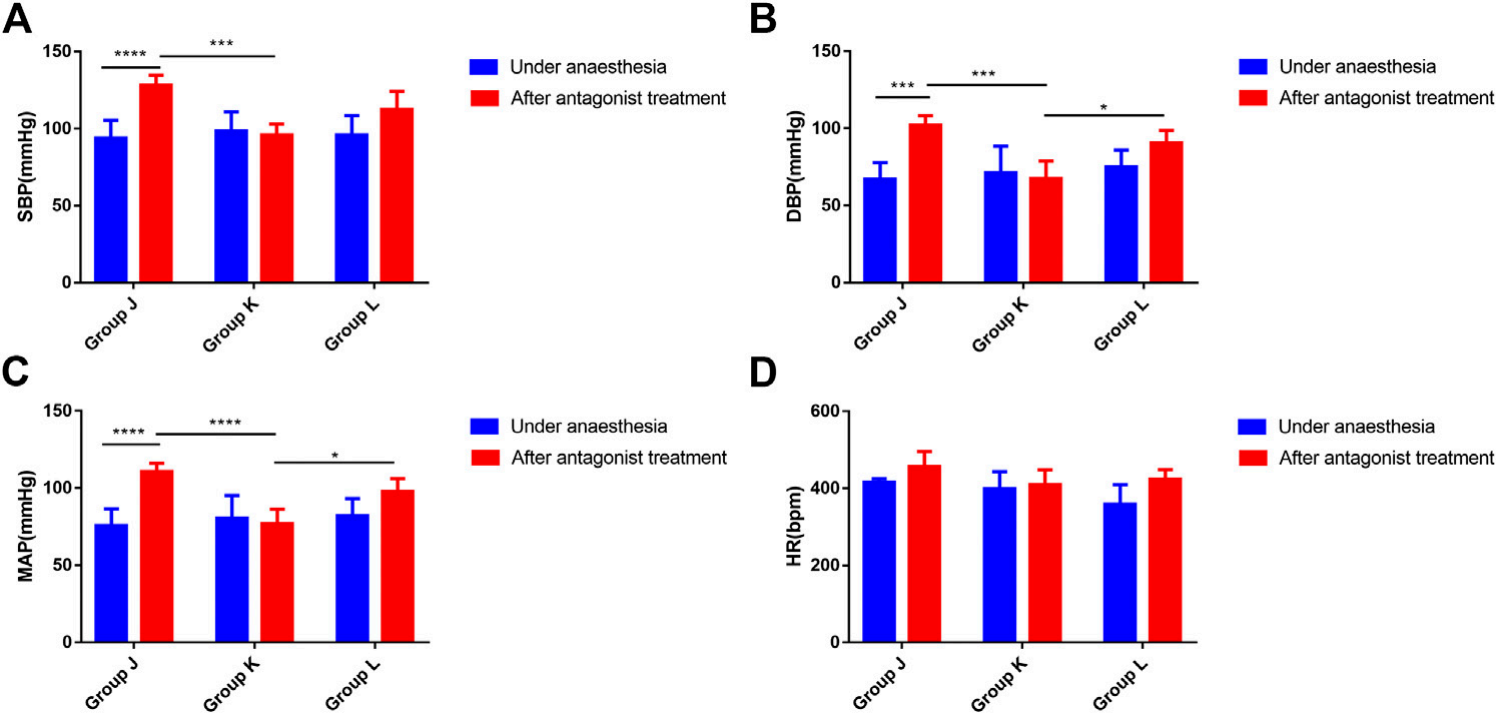
Effects on blood pressure and heart rate in rats after neostigmine or esmolol treatment **(A)**. Changes in SBP associated with the neostigmine or esmolol treatment; **(B)**. Changes in DBP associated with the neostigmine or esmolol treatment; **(C)**. Changes in MAP associated with the neostigmine or esmolol treatment; **(D)**. Changes in HR associated with the neostigmine or esmolol treatment. Error bars represent the mean ± SD, and the comparison was performed using two-way ANOVA. SBP, systolic blood pressure; DBP, diastolic blood pressure; MAP, mean arterial pressure; HR, heart rate. Under anesthesia, the blood pressure and heart rate under anesthesia. After antagonists, the highest blood pressure and heart rate within 40 min of using antagonists. Group J received phenylephrine and atropine simultaneously followed by intravenous saline treatment 1 min later. Groups K and L received phenylephrine and atropine simultaneously followed by 0.15 mg/kg neostigmine and 1.0 mg/kg esmolol treatment 1 min later. *p < 0.05, **p < 0.01. N = six.

Under anesthesia, no significant differences in SBP, DBP, MAP, or heart rate were observed among the groups (ANOVA, F = 0.2181, *p* = 0.8065; ANOVA, F = 0.57, *p* = 0.5773; ANOVA, F = 0.4008, *p* = 0.6767; ANOVA, F = 3.356, *p* = 0.0624) (Figure 3). Compared with the normal saline group, SBP, DBP and MAP significantly decreased in the neostigmine group (ANOVA, 95% CI = 12.54–51.72 mmHg, *p* = 0.0002; ANOVA, 95% CI = 13.69–55.09 mmHg, *p* = 0.0002; ANOVA, 95% CI = 14.58–52.7 mmHg, *p* < 0.0001). The mean heart rate decreased in the neostigmine group (Figure 3). However, the variations in heart rate of the neostigmine group were not significantly different compared with the normal saline group (*t* test, 95% CI = −131.9 to 33.88 bpm, *p* = 0.2174) (Table 2). Compared with the normal saline group, SBP, DBP, MAP, and HR were not significantly different in the esmolol group (ANOVA, 95% CI = −3.817–35.36 mmHg, *p* = 0.2119; ANOVA, 95% CI = −9.34–32.06 mmHg, *p* = 0.7619; ANOVA, 95% CI = −6.226–31.89 mmHg, *p* = 0.4625; ANOVA, 95% CI = −36.28 to 100.9 bpm, *p* = 0.9038) (Figure 3). Compared with the normal saline group, the heart rate increased in the esmolol group, and the variations in heart rate were not significantly different (*t* test, 95% CI = −59.63 to 134.3 bpm, *p* = 0.4110) (Table 2). The heart rate decreased to 409.50 ± 38.96 bpm after neostigmine treatment, which was lower than in the normal saline group (456.5 ± 39.1) and the esmolol group (424.17 ± 24.80) (Figure 3).

**TABLE 2:** Variations in BP and HP after neostigmine treatment in rats.

	SBP (mmHg)	DBP (mmHg)	MAP (mmHg)	HR (bpm)
Group J	34.44 ± 16.03	35.02 ± 15.98	34.83 ± 14.86	40.33 ± 86.46
Group K	−7.65 ± 6.97^***^	−11.53 ± 13.23^***^	−10.24 ± 11.04^***^	−8.67 ± 28.74
Group L	10.11 ± 16.55*	6.73 ± 16.21*	7.82 ± 16.12*	77.67 ± 62.36

Error bars represent the mean ± SD, and the comparison was performed using a *t* test. SBP, systolic blood pressure; DBP, diastolic blood pressure; MAP, mean arterial pressure; HP, heart rate. Group J received phenylephrine and atropine simultaneously followed by intravenous saline treatment 1 min later. Groups K and L received phenylephrine and atropine simultaneously followed by 0.15 mg/kg neostigmine and 1.0 mg/kg esmolol treatment 1 min later, respectively. ***p < 0.001, compared with the normal saline group. N = six.

## Discussion

Based on clinical observations and animal experiments, we found hazardous effects of the combination of topical phenylephrine and intravenous atropine treatment on blood pressure and heart rate. In pediatric patients, five to 25 min after intravenous atropine treatment SBP and DBP were more than 20% higher than the baseline blood pressure level. Acute elevated blood pressure (SBP, DBP, or MAP over 20% of the baseline level) during the intraoperative period can be considered a hypertension emergency (Goldberg and Larijani, 1998). A hypertensive emergency is a serious life-threatening clinical syndrome with progressive acute impairment of the heart, brain, kidney, and other important target organs (Varon and Marik, 2008; Muiesan et al., 2015). In our cases, clinically high blood pressure and tachycardia commonly occurred in normotensive patients under anesthesia. Patients with a previous history of hypotension were prone to perioperative blood pressure fluctuations when using phenylephrine (Robertson, 1979). Hypertensive crizes were more likely to occur in patients with hypertension, especially when SBP was >180 mmHg or DBP was >110 mmHg (Aronson et al., 2002; Varon and Marik, 2008). More attention should be paid to such patients when phenylephrine instillation and intravenous atropine are used together. Animal experiments showed that the increase in both blood pressure and heart rate can be reduced by implementing longer time intervals between the administration of the two drugs. The maximum plasma levels from phenylephrine eyedrop administration are achieved within ten to 20 min after topical instillation (Kumar et al., 1985). Consistent with our findings from animal experiments, we suggest that using intravenous atropine after prolonged time periods (10 or 15 min after phenylephrine eye drops) could reduce the increase in blood pressure and heart rate. Tachycardia is a heart rate that exceeds the normal resting rate; it is a common event in patients under anesthesia, and it is often associated with hypertension. Intraoperative tachycardia was associated with poorer outcomes, such as increased mortality and likelihood of intensive care unit admission and prolonged hospital stay (Reich et al., 2002; Hartmann et al., 2003). When children are ten years old, the normal heart rate is 90 bpm and can vary from 70 to 110 bpm. Tachycardia in ten-year-old children is considered when the heart rate is over 90 bpm while the patient is under anesthesia. Among our clinical cases, the heart rate began to increase significantly 10 min after administration of medication and the increase lasted for 20 min. The mean heart rate was over 90 bpm from ten to 30 min after intravenous atropine treatment. The tachycardia lasted for 20 min. The animal experiments showed that the heart rate increased by 20.50 ± 40.32 to 77.17 ± 57.72 bpm after the concurrent use of atropine and phenylephrine at different interval times.

Hypertensive emergencies usually require the intravenous administration of antihypertensive drugs (Kurnutala et al., 2014). Esmolol is a super short-acting selective beta antagonist. It can be used when the blood pressure and heart rate increase during surgery. Atropine could act competitively on M receptor with acetylcholine and thus heart rate could accelerate by inhibition of the excitatory tone of the vagal nerve. Neostigmine reversibly inhibits the activity of cholinesterase, thus increasing the concentration of acetylcholine at the receptor site in the body, strengthening and prolonging the effect of acetylcholine. Animal experiments have shown that neostigmine could significantly reduce blood pressure after they were increased due to the combined use of intravenous atropine treatment and phenylephrine instillation. Following the use of neostigmine, SBP and DBP decreased 7.65 ± 6.97 and 11.53 ± 13.23 mmHg, respectively. The heart rate decreased to 409.50 ± 38.96 bpm after neostigmine treatment, which was lower than that of the normal saline group after antagonist treatment. Meanwhile, SBP, DBP, MAP and HR did not decrease significantly in the esmolol group compared with the normal saline group. In our clinical cases, reducing the blood pressure and controling the heart rate by increasing the depth of anesthesia, increasing analgesia, and using beta blockers were difficult. We found that neostigmine could transiently improve the hemodynamic state while strengthening and prolonging the effect of acetylcholine. Clinical observations and animal experiments have indicated that neostigmine could distinctly reduce blood pressure and heart rate. Atropine may have played a leading role in this effect. In conclusion, neostigmine may be a safe and effective choice for reducing the blood pressure and heart rate in pediatric patients following the combined use of intravenous atropine treatment and phenylephrine instillation.

However, our clinical study had several limitations. A small sample size and the single-centred retrospective study are the major limitations of our study. In addition, this study only focused on pediatric patients and had a limited number of patients (more boys), resulting in considerably biased clinical features. A further retrospective study is needed in multiple centers with a large sample to observe the adverse cardiovascular effects of phenylephrine eye drops combined with intravenous atropine in patients.

## Conclusion

We suggest the following recommendations for the clinical use of atropine and phenylephrine in pediatric patients:

1. The combined use of phenylephrine and atropine eye drops should be avoided.
2. Intravenous atropine can be used only for blunting OCR after a certain time interval, usually more than 10 min, after using phenylephrine eye drops.
3. The patient can be rescued with neostigmine if the concurrent use of intravenous atropine and phenylephrine eye drops results in clinically significant high blood pressure and heart rate.

To avoid serious adverse reactions, the following points should be noted for the clinical use of compound tropicamide eye drops:

1. Patients must be asked about their medical history and drug allergy history. Patients with cardiovascular disease should be treated with caution.
2. The nasolacrimal passage should be compressed for 60 s immediately following topical administration of compound tropicamide eye drops to minimize drainage.
3. Patients should be closely observed for systemic adverse reactions.

## Data Availability Statement

The original contributions presented in the study are included in the article/Supplementary Material, further inquiries can be directed to the corresponding authors.

## Ethics Statement

The studies involving human participants were reviewed and approved by Medical Ethics Committee of Zhongshan Ophthalmic Center, Sun Yat-sen University. Written informed consent from the participants' legal guardian/next of kin was not required to participate in this study in accordance with the national legislation and the institutional requirements. The animal study was reviewed and approved by The laboratory animal ethics committee of Drug Evaluation Center, Guangzhou Boji Pharmaceutical Biotechnology Co., Ltd (No. 00201878).

## Author Contributions

WG conceived and designed the study and provided the clinical data. YG, YW, and HZ performed the animal experiments. YG, QL, SZ and JT performed the analyses. YW, JP, and XY conducted the analyses. QL and YD wrote the paper. WG and WC reviewed and edited the manuscript. All authors read and approved the manuscript.

## Funding

This work was supported by the Science and Technology Program of Guangzhou of Wei Chi (No. 201804010415).

## Conflict of Interest

The authors declare that the research was conducted in the absence of any commercial or financial relationships that could be construed as a potential conflict of interest.
